# Supplementary material for: Chloroquine-Induced DNA Damage Synergizes with Nonhomologous End Joining Inhibition to Cause Ovarian Cancer Cell Cytotoxicity
Source: Int J Mol Sci. 2022 Jul 7;23(14):7518. doi: 10.3390/ijms23147518 (PMC9323666; doi:10.3390/ijms23147518)
Supplement: Supplementary file 1 [file ijms-23-07518-s001.zip › ijms-1762939-supplementary.pdf]

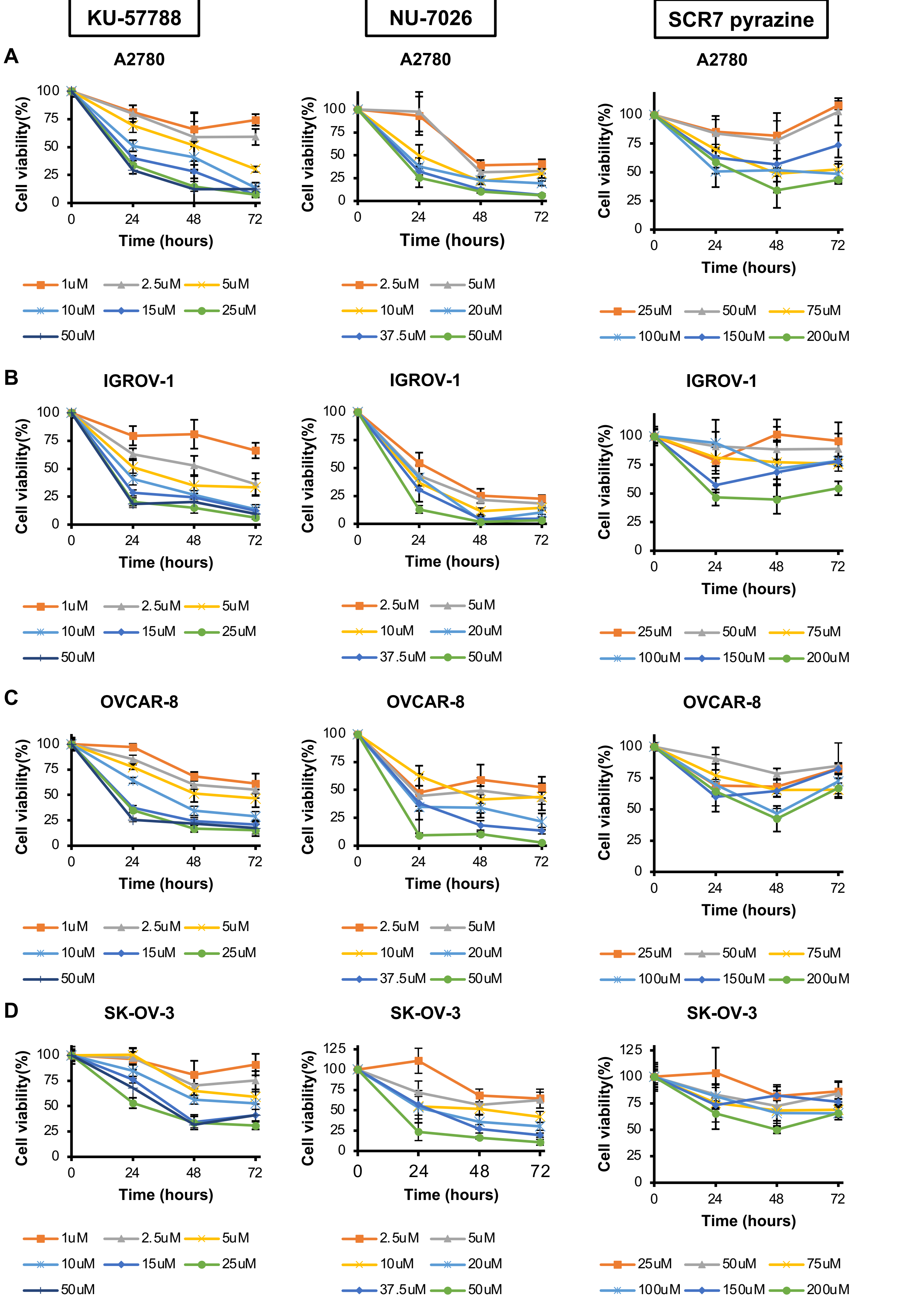

Figure S1. Effect of NHEJi on OCCLs proliferation. Cell proliferation after treatment with the indicated doses of KU-57788, NU-7026 or SCR7 pyrazine for 24, 48 and 72 h of (A) A2780, (B) IGROV-1, (C) OVCAR-8 and (D) SK-OV-3 cell lines.

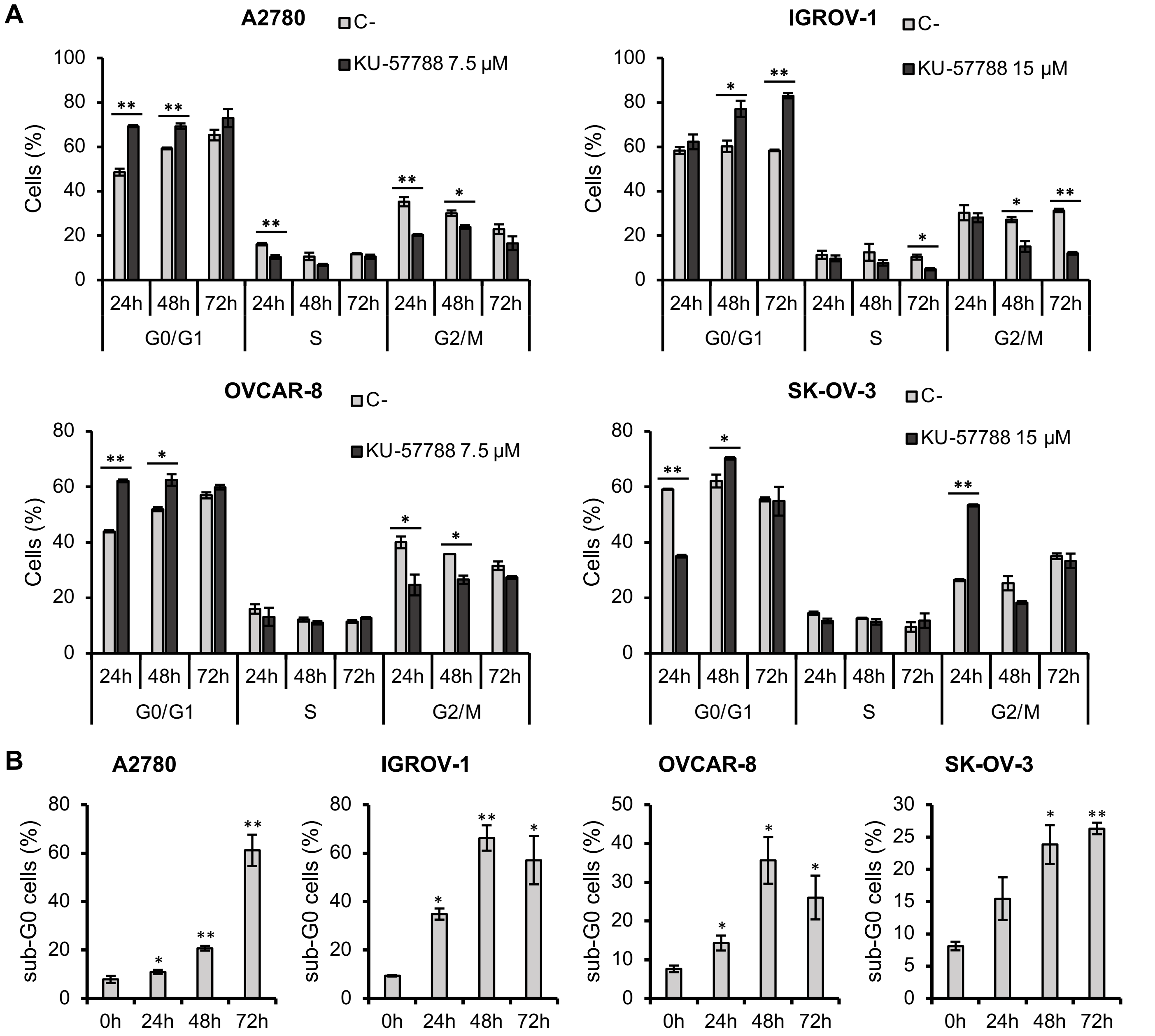

Figure S2. Effect of KU-57788 on cell cycle distribution in OCCLs. Cell cycle distribution of live OCCLs (excluding the sub-G0 population) after treatment with the indicated doses of KU-57788 for 24, 48 and 72 h. Data are the mean of three independent experiments. Error bars represent the SD (**p < 0.01, *p < 0.05). (B) Percentage of death cells after 24,48 or 72 h of treatment with KU-57788.

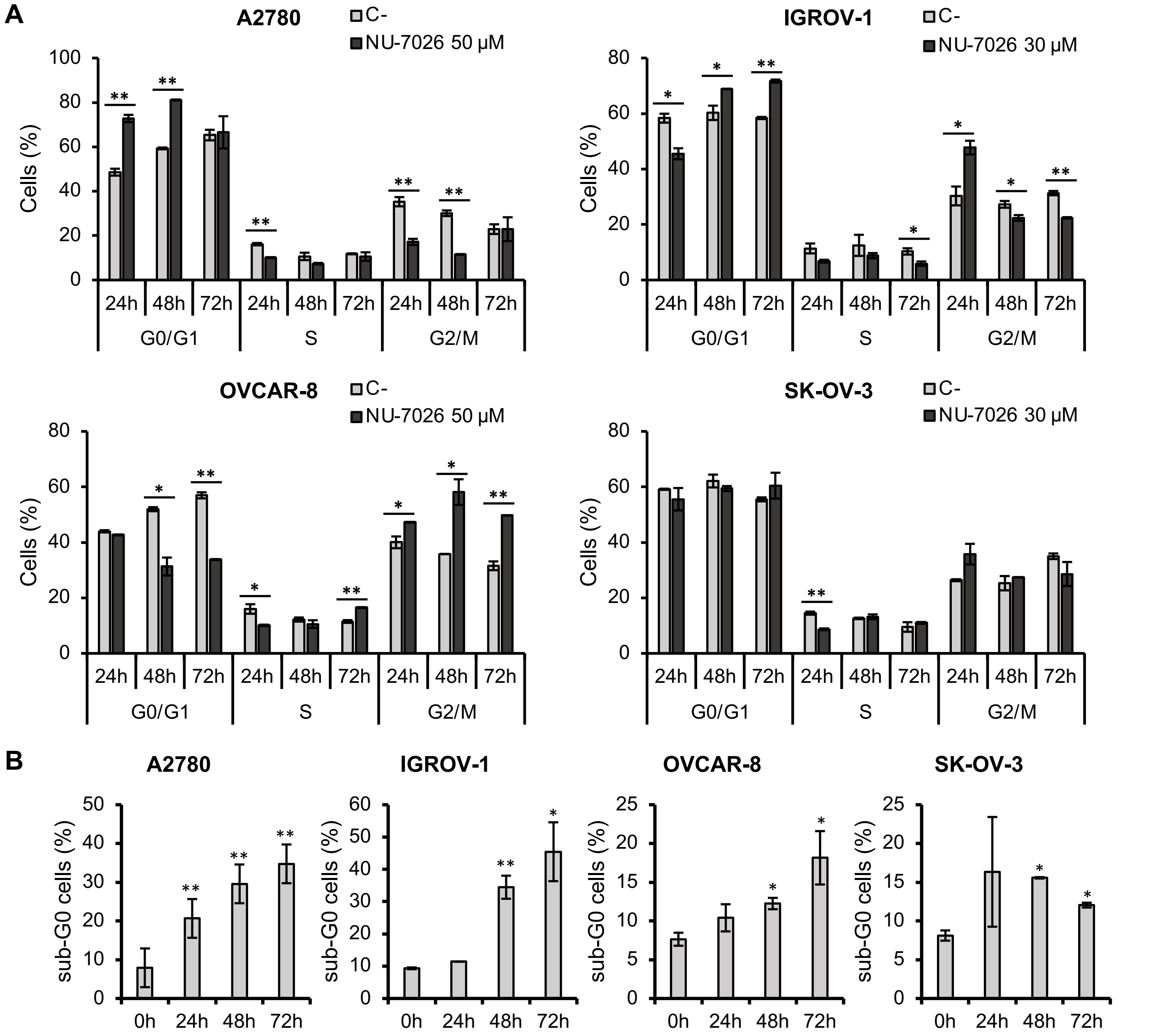

Figure S3. Effect of NU-7026 on cell cycle distribution in OCCLs. Cell cycle distribution of live OCCLs (excluding the sub-G0 population) after treatment with the indicated doses of NU-7026 for 24, 48 and 72 h. Data are the mean of three independent experiments. Error bars represent the SD (**p < 0.01, *p < 0.05). (B) Percentage of death cells after 24,48 or 72 h of treatment with NU-7026.

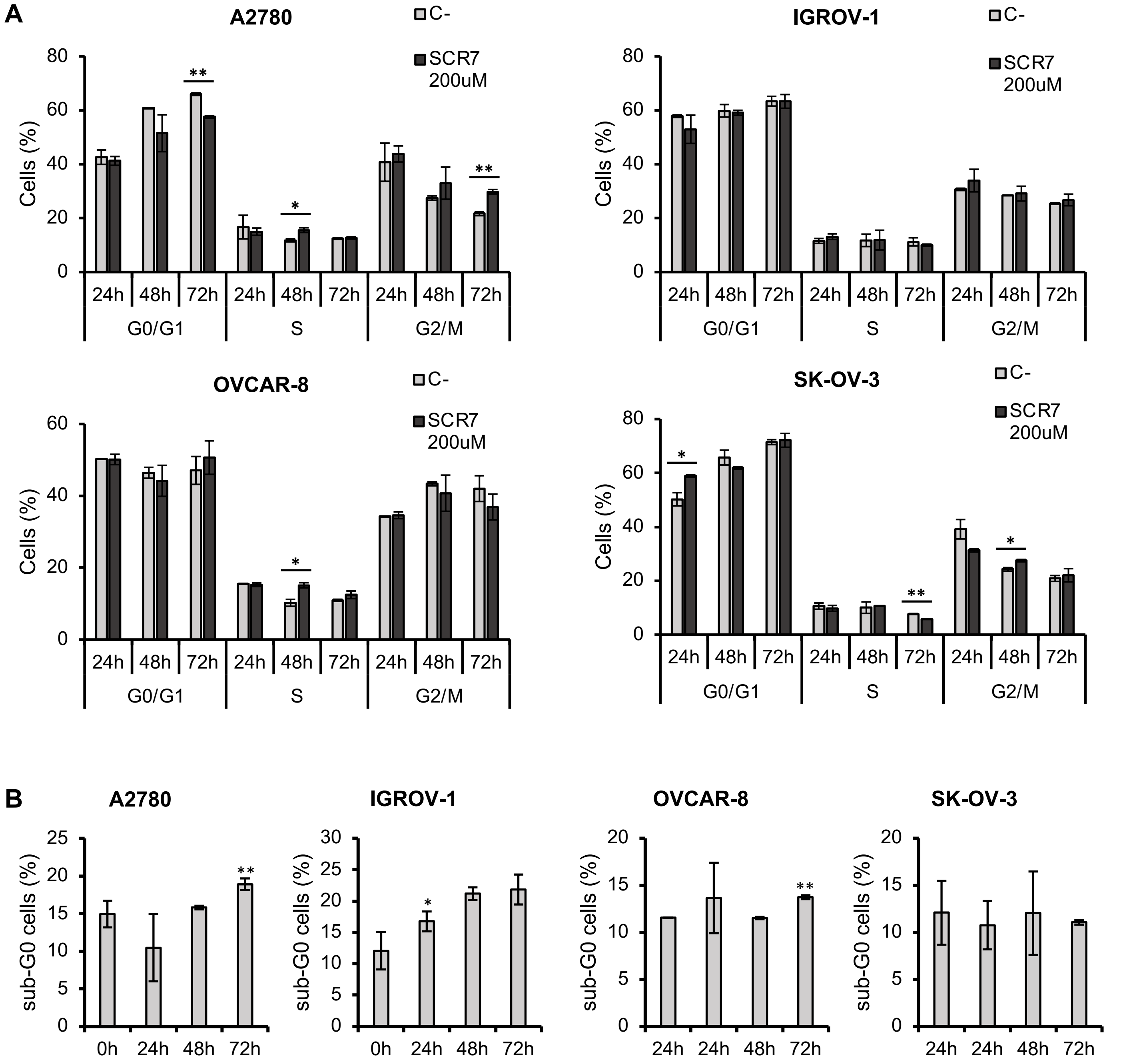

Figure S4. Effect of SCR7 pyrazine on cell cycle distribution in OCCLs. (A) Cell cycle distribution of live OCCLs (excluding the sub-G0 population) after treatment with the indicated doses of SCR7 for 24, 48 and 72 h. Data are the mean of three independent experiments. Error bars represent the SD (**$p < 0.01$, *$p < 0.05$). (B) Percentage of death cells after 24,48 or 72 h of treatment with SCR7.

OVCAR-8

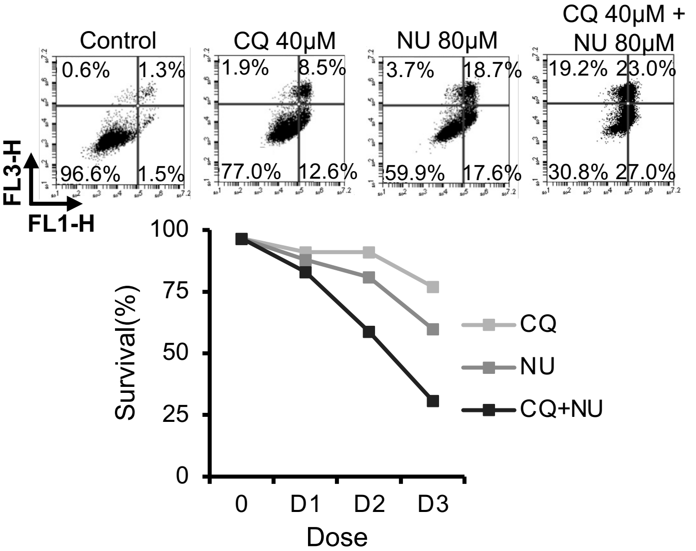

Dose		D1	D2	D3	D1	D2	D3	D1	D2	D3
CQ (µM)	-	20	30	40	-	-	-	20	30	40
NU (µM)	-	-	-	-	40	60	80	40	60	80
Survival (%)	96.6	90.9	91.0	77.0	88.0	80.9	59.9	83.0	58.7	30.8
Fa	0.03	0.09	0.09	0.23	0.12	0.19	0.40	0.17	0.41	0.69
CI	-	-	-	-	-	-	-	**1.34**	**1.04**	**0.76**

A2780

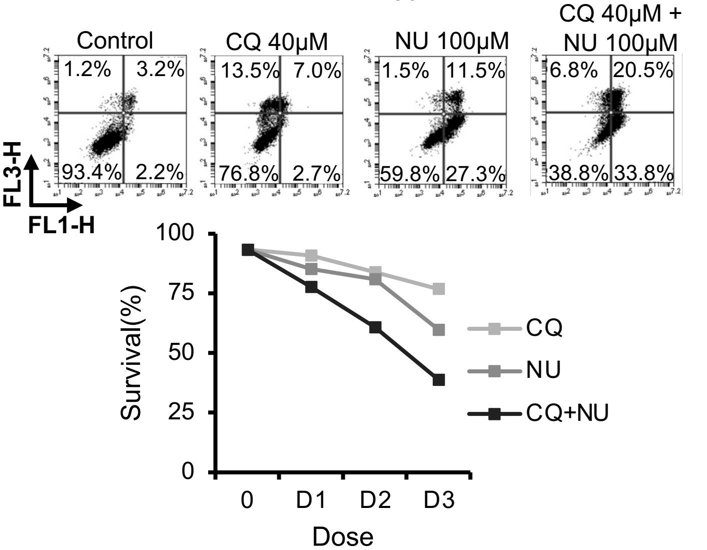

Dose		D1	D2	D3	D1	D2	D3	D1	D2	D3
CQ (µM)	-	20	30	40	-	-	-	20	30	40
NU (µM)	-	-	-	-	50	75	100	50	75	100
Survival (%)	93.4	90.9	84.0	76.8	85.2	81.0	59.8	77.7	60.8	38.8
Fa	0.07	0.09	0.16	0.23	0.15	0.19	0.40	0.22	0.39	0.61
CI	-	-	-	-	-	-	-	**1.03**	**0.88**	**0.66**

SK-OV-3

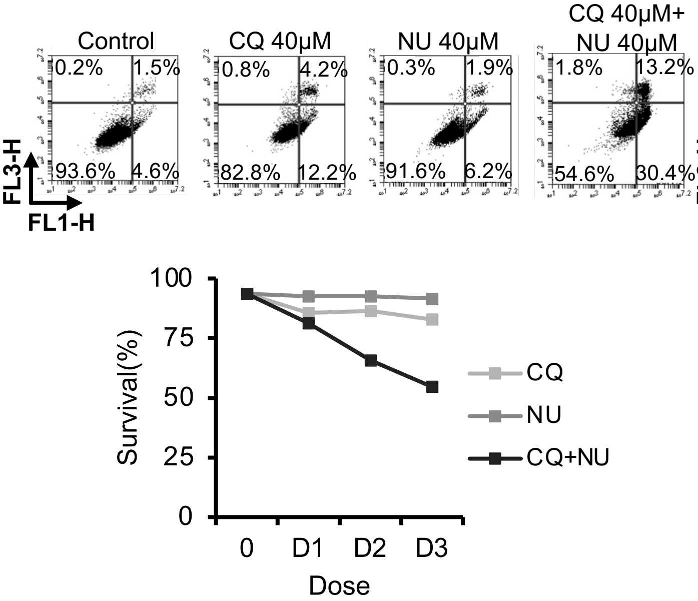

Dose		D1	D2	D3	D1	D2	D3	D1	D2	D3
CQ (µM)	-	20	30	40	-	-	-	20	30	40
NU (µM)	-	-	-	-	20	30	40	20	30	40
Survival (%)	93.6	85.6	86.3	82.8	92.7	92.5	91.6	81.4	65.8	54.6
Fa	0.06	0.14	0.14	0.17	0.07	0.08	0.08	0.19	0.34	0.45
CI	-	-	-	-	-	-	-	**0.28**	**0.02**	**0.005**

IGROV-1

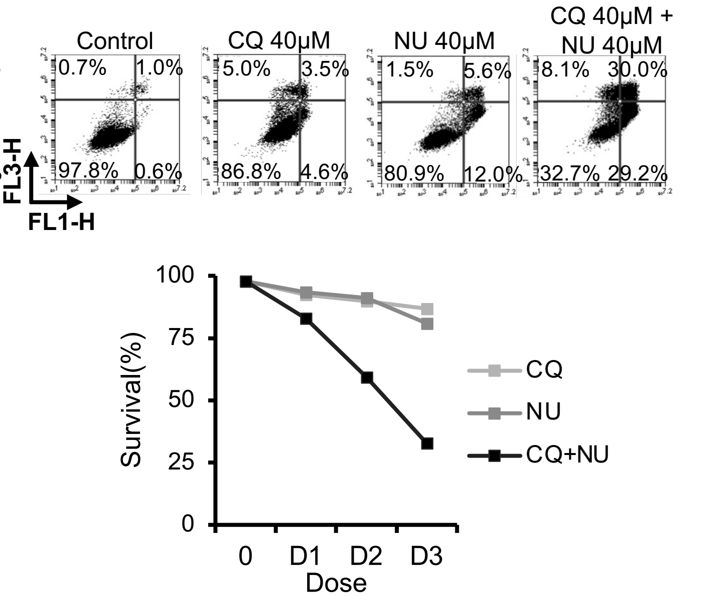

Dose		D1	D2	D3	D1	D2	D3	D1	D2	D3
CQ (µM)	-	20	30	40	-	-	-	20	30	40
NU (µM)	-	-	-	-	20	30	40	20	30	40
Survival (%)	97.8	92.3	89.8	86.8	93.5	91.1	80.9	82.9	59.2	32.7
Fa	0.02	0.08	0.10	0.13	0.07	0.09	0.19	0.17	0.41	0.67
CI	-	-	-	-	-	-	-	**0.84**	**0.49**	**0.30**

Figure S5. Synergistic effect of chloroquine and NU-7026 in OCCLs. Cells were exposed for 72 h to the indicated concentrations of NU and CQ at a constant ratio and the percentage of apoptotic cells were assessed by flow cytometry (after cell staining with annexin V and propidium iodide). CI values, calculated using Compusyn Software, are shown.

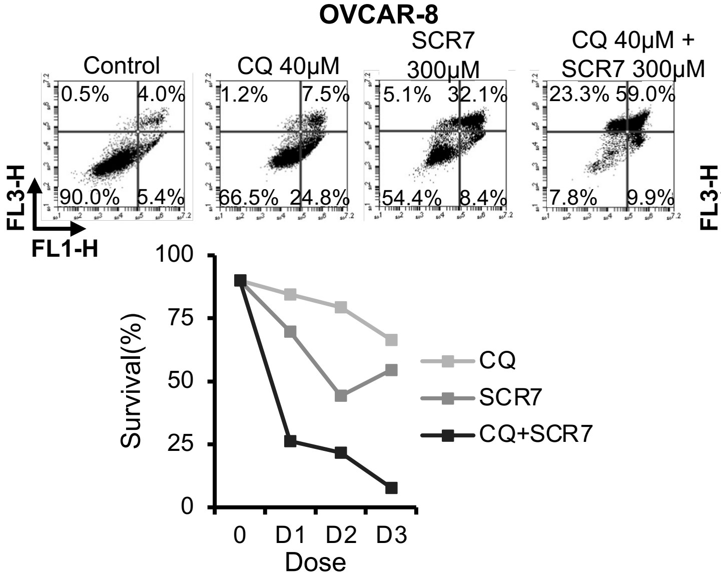

Dose		D1	D2	D3	D1	D2	D3	D1	D2	D3
CQ (µM)	-	20	30	40	-	-	-	20	30	40
SCR7 (µM)	-	-	-	-	150	225	300	150	225	300
Survival (%)	90.0	84.4	79.4	66.5	69.8	44.2	54.4	26.2	21.7	7.8
Fa	0.10	0.16	0.21	0.34	0.30	0.56	0.46	0.74	0.78	0.92
CI	-	-	-	-	-	-	-	**0.34**	**0.42**	**0.21**

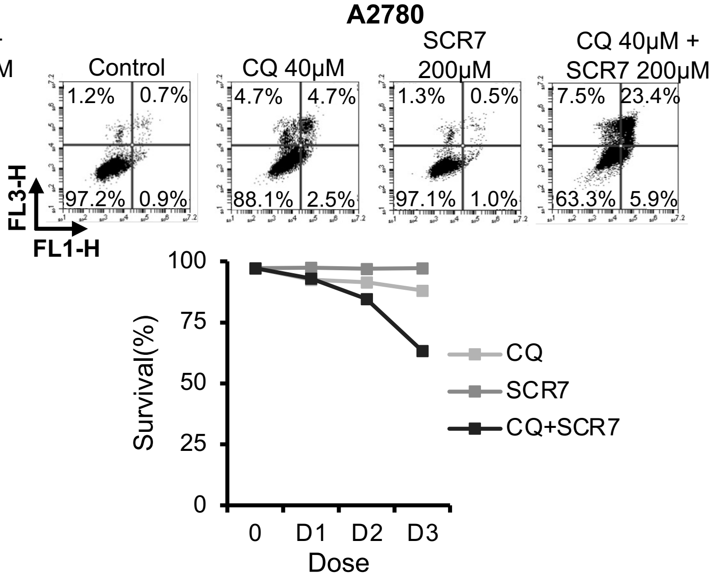

Dose		D1	D2	D3	D1	D2	D3	D1	D2	D3
CQ (µM)	-	20	30	40	-	-	-	20	30	40
SCR7 (µM)	-	-	-	-	100	150	200	100	150	200
Survival (%)	97.2	92.5	91.5	88.1	97.4	97.0	97.1	93.1	84.6	63.3
Fa	0.03	0.08	0.09	0.12	0.03	0.03	0.03	0.07	0.15	0.37
CI	-	-	-	-	-	-	-	**1.07**	**0.45**	**0.12**

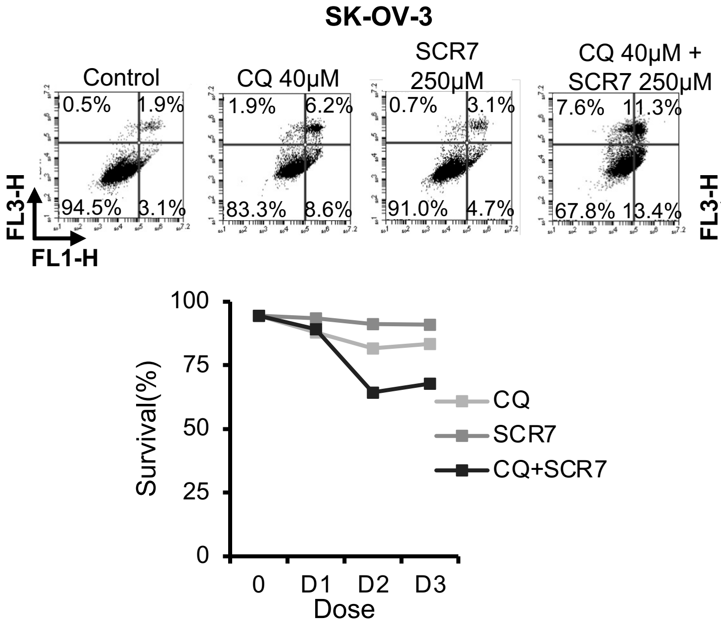

Dose		D1	D2	D3	D1	D2	D3	D1	D2	D3
CQ (µM)	-	20	30	40	-	-	-	20	30	40
SCR7 (µM)	-	-	-	-	125	187.5	250	125	187.5	250
Survival (%)	94.5	87.9	81.7	83.3	93.4	91.1	91.0	89.1	64.4	67.8
Fa	0.06	0.12	0.18	0.17	0.07	0.09	0.09	0.11	0.36	0.32
CI	-	-	-	-	-	-	-	**1.75**	**0.19**	**0.32**

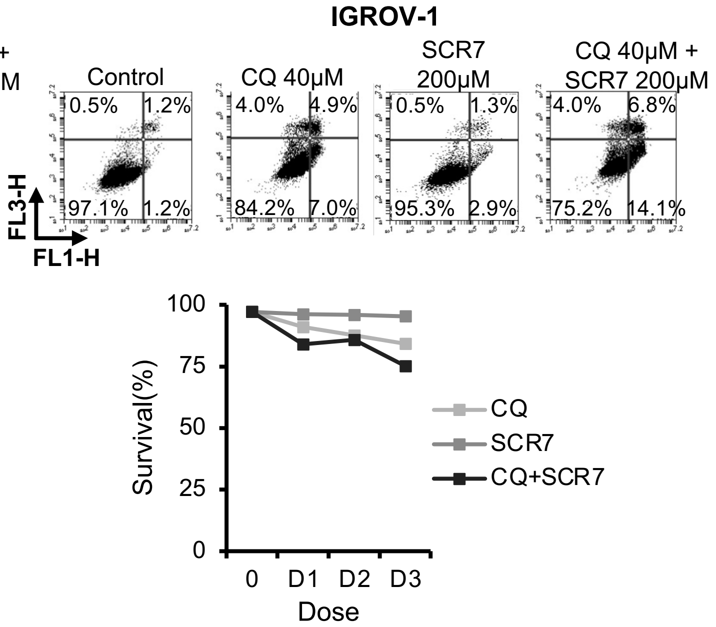

Dose		D1	D2	D3	D1	D2	D3	D1	D2	D3
CQ (µM)	-	20	30	40	-	-	-	20	30	40
SCR7 (µM)	-	-	-	-	100	150	200	100	150	200
Survival (%)	97.1	90.9	87.6	84.2	96.3	95.9	95.3	84.1	85.8	75.2
Fa	0.03	0.09	0.12	0.16	0.04	0.04	0.05	0.16	0.14	0.25
CI	-	-	-	-	-	-	-	**0.50**	**0.88**	**0.54**

Figure S6. Synergistic effect of chloroquine and SCR7 pyrazine in OCCLs. Cells were exposed for 72 h to the indicated concentrations of SCR7 and CQ at a constant ratio and the percentage of apoptotic cells were assessed by flow cytometry (after cell staining with annexin V and propidium iodide). CI values, calculated using Compusyn Software, are shown.

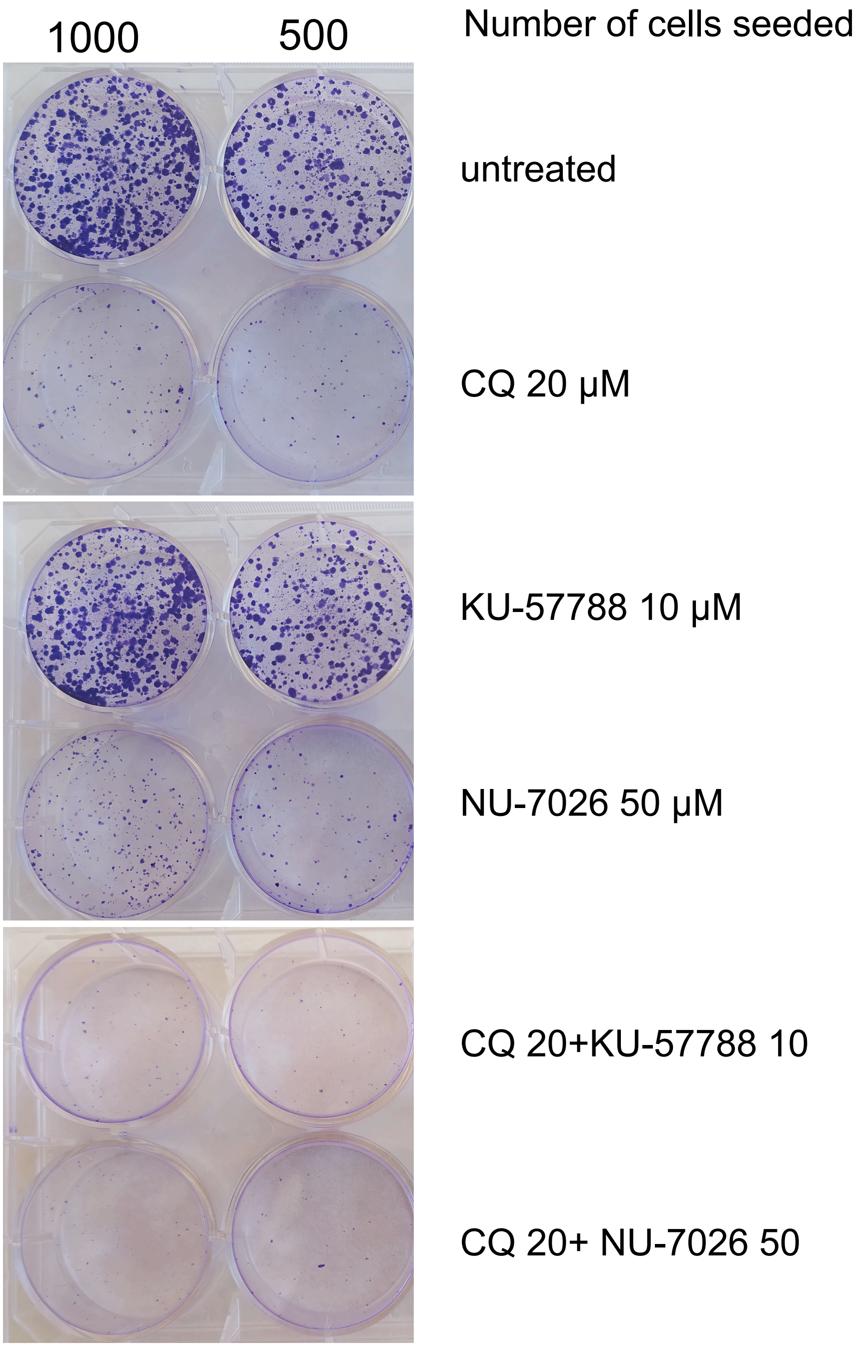

Figure S7. Clonogenic assays. A2780 cells were seeded in 6 well plates and 24 h later they were incubated in the presence of the indicated compounds for 72 hours. Cells were then washed with PBS and incubated in complete RPMI1640 medium for 5 days. Colonies were fixed with 4% paraformaldehyde and stained with crystal violet.
